# Direct and Indirect Neurotoxic Potential of Metal/Metalloids in Plants and Fungi Used for Food, Dietary Supplements, and Herbal Medicine

**DOI:** 10.3390/toxics9030057

**Published:** 2021-03-16

**Authors:** Peter S. Spencer, Valerie S. Palmer

**Affiliations:** 1 Department of Neurology, Oregon Health & Science University, Portland, OR 97239-3098, USA; palmerv@ohsu.edu; 2 Oregon Institute of Occupational Health Sciences, Oregon Health & Science University, Portland, OR 97239-3098, USA

**Keywords:** soil and water pollution, heavy metals, morels, grasspea, cassava, neurodegeneration

## Abstract

Plants and mushrooms bioconcentrate metals/metalloids from soil and water such that high levels of potentially neurotoxic elements can occur in cultivated and wild species used for food. While the health effects of excessive exposure to metals/metalloids with neurotoxic potential are well established, overt neurological disease from prolonged ingestion of contaminated botanicals has not been recognized. However, the presence of metal elements may affect levels of botanical neurotoxins in certain plants and mushrooms that are established causes of acute and chronic neurological disease.

## 1. Introduction

Plants and mushrooms that are/are not used for food can sequester metals/metalloids drawn from soil and water that have the potential for human neurotoxic effects. Although this property of botanicals is exploited for the bioremediation of metal-contaminated soils, the possibility of toxic/neurotoxic effects in the consumer of metal-concentrating plants and mushrooms has been rarely addressed. This requires a synthesis of mycology, botany, and toxicology, not only of the human and mammal but also of the plant and mushroom.

Three scenarios are considered here: certain metals contained in plants and mushrooms used for food pose: (a) a direct threat to human neurological function and/or (b) an indirect threat because they can modulate the concentration of botanical compounds (hydrazine, dencichine, cyanogens) that by themselves pose a neurotoxic hazard to the consumer. Examples of the latter include certain plants (grasspea, cassava) and mushrooms (false morel), food use of which can precipitate neurotoxic disease.

## 2. Botanical Uptake of Metal Elements Required for Biological Function

The major elements in soil (>100 mg/kg), and minerals derived therefrom, include aluminum, calcium, carbon, iron, magnesium, nitrogen, oxygen, potassium, silicon, sodium, sulfur, and titanium. Other soil elements include barium, chlorine, manganese, phosphorus, strontium, and zirconium [1,2]. Common trace elements in soil (<100 mg/kg) comprise arsenic, chromium, cobalt, copper, lead, lithium, nickel, selenium, and zinc [1]. Whereas Ca, Fe, K, Mn, and P are required for normal human physiological function, prolonged excessive exposure to some of the listed elements (Al, As, Cu, Fe, Li, Pb), among others (Cd, Hg, Tl, Zn), can result in neurologic dysfunction or overt disease.

Soil goes through various stages of aging that result in changes in its chemistry and that of associated water. The resulting chemical environment is reflected in the organisms that feed on soil nutrients [1,2]. Fungi play a vital role in the soil environment by acting as a bridge between soil microbes and plants, thereby facilitating nutrient cycling and plant health and disease control [3]. Fungi are particularly effective in changing the solubility of metals by employing three major reaction types that change the speciation of metal complexes: reduction, methylation, and dealkylation [4]. Fungal hyphae connect with the root systems of plants, making the constant exchange of nutrients possible [5]. Certain metals are biologically active in mushrooms, including Ca, Co, Cu, Fe, K, Mg, Mn, Na, Ni, and Zn [6]. Fungi secrete a chelator that binds iron, solubilizing the element and allowing it to enter cells [7].

Twenty elements are considered essential for plant growth, including those that may derive from air (carbon, oxygen, hydrogen), air and soil (nitrogen), or soil alone, such as boron, calcium, chorine, copper, iron, magnesium, manganese, molybdenum, and zinc [8]. Certain fungi and plants can form a symbiotic relationship in which the mushrooms help plants acquire trace nutrients in exchange for carbon. Fungi specialize in concentrating elements and passing them along to plants. About 80% of plant roots participate in this type of relationship, meaning that almost all nutrients taken up by plants first transit through fungi [9]. Thus, the function of fungi is to accumulate essential elements (notably K, Na, and Zn) not only for mushroom growth but also for that of plants and, indirectly, for animals and humans that consume components as food [2].

## 3. Fungal Uptake of Metals/Metalloids with Neurotoxic Potential

While fungi absorb metals in soil required for their normal growth and pigmentation, they also take up and bioaccumulate soil metals that are present in concentrations that would be harmful to plants [4]. In turn, certain metals found in soils, often due to anthropogenic activities, can harm mushrooms by competing for binding with elements that fungi need for metabolism and reproduction [2,10]. In general, fungi adapt to the soil content of metals [11], as illustrated by arsenic and lead, but high levels of certain elements can perturb fungal growth and development [12]. The ability of mushrooms to accumulate heavy metals is relevant to their use by humans for food, nutrient supplementation, and mycomedicine.

Numerous studies have measured the concentration of various elements in mushrooms, including metals/metalloids with potential for human neurological disease. Arsenic concentrations in the fruit bodies of 37 common edible mushrooms ranged from >0.05 mg/kg to 146.9 mg/kg [13]. The ability to accumulate arsenic was found in mushrooms with saprotrophic nutrition (feeding on nonliving organic matter), including the Basidiomycetes families of Agaricaceae, Tricholomataceae, and Gasteromycetaceae. By contrast, arsenic accumulation was not detectable in xylophagous (rotting wood-feeding) or mycorrhizal (plant-fungus symbiotic) species of edible mushrooms. Forms of arsenic found in mushrooms include arsenobetaine, arsenate, arsenocholine, and unidentified compounds containing the trimethylarsonium ion [14,15]. Fresh fruit bodies reportedly contain about a tenfold lower arsenic level than dried specimens [13]. Studies from northwest Spain and Dhaka, Bangladesh, reported low mean concentrations of arsenic (0.27 and 0.51 mg/kg dry weight, respectively) in wild and cultivated mushrooms [16,17].

As with arsenic, widely divergent levels of lead have been found in fungi. Some reports do not clarify whether content refers to fresh fungal tissue or dry weight (dw). A study from Tuscany, Italy, reported a range of lead concentrations from 0.4 to 15.5 mg/kg in fungi and 22 to 51 mg/kg in soil [18]. Lead content ranged from 1.9 to 10.8 mg/kg in mushrooms collected from three sites in China [19] and between 0.4 and 36 mg/kg in Sweden [20]. A similar concentration range (0.60–11.4 mg/kg) was found in wild mushrooms collected in Turkey [21], while lower levels (up to 2.4 mg/kg) were found in species collected in parts of Germany, Macedonia, Greece, and Turkey [22,23,24,25]. High levels of lead were measured in wild mushrooms (76.00 ± 9.78 mg/kg) and edible species (6.46–27.33 mg/kg) sold in Nigeria [26,27].

Several other studies have examined the concentration of metals in fungi, including elements with neurotoxic potential. Reports focused on tissue mercury content [28,29] found high levels (4.9–22 mg kg dw) in edible *Boletus* species in the mercuriferous belt of southwestern China [30] and lower levels (2.28 mg/kg) in *B. edulis* (edible Penny Bun mushroom) collected in Croatia [31]. Cadmium and silver are also taken up by *B. edulis* [32]. Analysis of 14 wild edible mushrooms collected in Yunnan, China, identified high concentrations of manganese (13.5–113 mg/kg) and iron (67.5–843 mg/kg) [33]. Lead, cadmium, mercury, and selenium were found in 60 species of common edible mushrooms collected mainly in the province of Reggio Emilia, Italy [34]. Species of *Agaricus*, *Macrolepiota*, *Lepista*, and *Calocybe* accumulate a high content of cadmium and mercury, even in unpolluted areas, but the concentration of these metals increases considerably in heavily polluted sites, such as in the vicinity of both working and abandoned metal smelters or inside cities [35]. Blanching and pickling edible mushrooms reduce their metal content [35].

A recent article analyzed 200 European publications (published between 2001 and 2016) that describe the concentration of selected elements in mushrooms [36]. Papers dealing with elements such as Cd, Cu, Fe, Pb, and Zn originated primarily from Turkey, Poland, Spain, and the Czech Republic. Many studies underlined the need to assess the risk to human health arising from the consumption of mushrooms taken from various contaminated habitats because polluted soils and water directly impact the concentration of elements in mushrooms. Those with a high lead content were collected from soils impacted by former metallurgical and mining activities. For example, in Příbram, Czech Republic, the upper soil layer had a lead concentration of 36,234 mg/kg, while the stipe of *B. edulis* growing in this area contained 165 mg/kg dw [37]. A copper concentration of 427 mg/kg dw was measured in samples of *B. edulis* collected near a copper smelter in Norway [38], while high concentrations of lead (11,460 mg/kg dw), manganese, and copper were measured in *Lepista (Clitocybe) nuda* (edible Wood Blewit mushroom) collected from the Eskişehir forest area of Turkey [39]. Many years of traffic pollution were blamed for the very high levels of iron (9685 mg/kg dw) in *Omphalotus olearius* (Jack-O’-Lantern mushroom), a poisonous xylophagous fungal species taken from the forest along the Balıkesir-Manisa highway in Turkey [40]. Other studies linked vehicular pollution to the lead content of certain fungal species collected near heavily trafficked roads [41]. The ability of mushrooms, such as *Pleurotus* species, to biosorb heavy metals has important applications for remediation of polluted soil and water of industrial origin [12]. Uptake of heavy metals by the mycelia of *P. ostreatus* (Oyster mushroom) increased proportionally to their concentration in the medium on which the fungus was grown [42].

Botanicals contain polyvalent phytic acid, which can bind bi- and trivalent cations of various elements. At neutral pH, metal binding to phytic acid corresponds to Cu > Zn > Ni > Co > Mn > Fe > Ca [43]. The cap of mushrooms produces stress-related factors (metallothionein) that govern the uptake of metal ions [44]. Cysteine-rich oligopeptides (phytochelatin family) bind a large fraction of cadmium in the caps of *B. edulis* when the edible mushroom is exposed to excess metals [45]. Fungi also bisorb and sequester heavy metals via melanin, a negatively charged hydrophobic pigment formed by the polymerization of indolic and phenolic compounds. Experiments with melanin extracted from *Armillaria cepistipes* (Ringless Honey Mushroom) revealed a differential metal affinity, namely Pb^2+^ *>* Cr^3+^ *>* Ni^2+^ *>* Cd^2+^ *>* Zn^2+^ *>* Ca^2+^—with an extreme preference for Pb^2+^ (80% removal) over the essential metals (0% and 12% removal for Ca^2+^ and Zn^2+^, respectively) [46]. Fungal melanin production can be both constitutive and facultative, production increasing according to environmental stressors, such as UV radiation, drying, high concentrations of salts, heavy metals, and radionuclides [47]. Melanized fungi are thus candidates for soil and water bio(myco)remediation [48].

## 4. Heavy Metals in Mushrooms with Potential Neurotoxicity

The True Morel *Morchella esculenta*, a facultative mycorrhizal mushroom widely prized by gourmets, illustrates the potential human health threat of consuming mushrooms that bioaccumulate metals from contaminated soil. In the USA, lead arsenate (PbHAsO4) was widely used as a moth pesticide from the late 1800s, replaced by DDT in the 1950s, and banned from use in fruit orchards in 1988. However, the potentially neurotoxic elements (Pb, As) persist in the topsoils on which morels grow. A study of 29 abandoned apple orchards in the northeastern USA revealed a range of lead and arsenic in soil (19.20–2450 and 3.08–244.00 mg/kg, respectively) and in the fruit bodies of *M. esculenta* (0.5–13.00 and 0.15–2.85 mg/kg, respectively) growing on polluted soils. The respective concentrations were statistically associated (r = 0.81) for lead and arsenic in soil, and for soil and morel lead content (r = 0.94, r = 0.57, respectively). Almost all (94%) of the arsenic stored in morel tissues was in the inorganic form, and the levels of the two elements in morel fruitbodies were considered to pose a human health risk [49]. A mycologist who consumed *M. esculenta* collected from apple orchards had elevated levels of urinary arsenic and lead; he complained of symptoms consistent with sensory (arsenic) neuropathy that resolved following chelation therapy [50].

**Figure 1:**
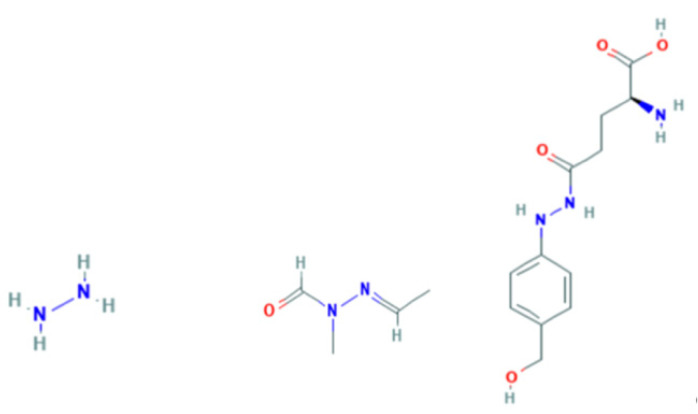
Monomethylhydrazine (**left**), gyromitrin (**center**), and agaritine (**right**). Source: PubChem, National Institutes of Health National Library of Medicine https://pubchem.ncbi.nlm.nih.gov/, accessed on 14 March 2021.

False Morel mushrooms, such as *Gyromitra esculenta,* a poisonous species that is nevertheless eaten by some, contain gyromitrin (acetaldehyde *N*-methyl-*N*-formyl-hydrazone) (Figure 1, center). While the function of fungal gyromitrin is unknown, hydrazones can form complexes with metals, such as Ni^2+^, Cu^2+^, Zn^2+^, and Cd^2+^ [51]. The principal metabolite of gyromitrin, monomethylhydrazine (MMH) (Figure 1, left), is an acutely neurotoxic compound that interferes with pyridoxine utilization by both glutamic acid decarboxylase and *γ*-aminobutyric acid (GABA) transaminase, leading to decreased concentrations of the inhibitory neurotransmitter GABA in the brain and consequent induction of seizures [52]. Levels of MMH in *G. esculenta* vary and can be reduced by prolonged desiccation [53]. Consumption of the False Morel *G. gigas* collected from soils near a closed lead mine in the French Alps has recently been linked to a focus of amyotrophic lateral sclerosis; while the authors did not measure lead levels in local False Morels, they attributed induction of neurodegenerative disease to the genotoxic properties of MMH [54].

Hydrazine is a reducing agent that is used industrially to reduce metal salts and oxides to pure metals. Phenylhydrazines, notably agaritine (*N*2-(γ-L-glutamyl)-4-hydroxymethylphenylhydrazine) (Figure 1, right), are found in *Agaricus* mushrooms. Concentrations range from 200 to 500 mg agaritine/kg fresh weight in cultivated species of the universally eaten *A. biporus* (Button Mushroom). Wild samples of *A. elvensis* have been reported to contain up to 10,000 mg agaritine/kg fresh weight [55,56]. Heavy metals (mg/kg) in the fruiting bodies of *A. bisporus* growing wild in Poland included: Cd, 0.68–6.14; Cr, 0.38–6.93; Cu, 1.90–101.71; Fe, 33.01–432.24; Mn, 2.86–387.43; Ni, 0.20–3.09; Pb, 0.98–42.83, and Zn, 31.87–124.84 [57]. Samples collected near Kermanshah City, Iran, contained levels of arsenic (mean 65.23 ± 13.57 mg/kg) and zinc (mean 66.23 ± 2.80 mg/kg) that exceeded the maximum permissible limit [58].

## 5. Plant Uptake of Metals/Metalloids with Neurotoxic Potential

The presence of heavy metals in plants used for food and their associated human health threats have been comprehensively reviewed by Rai and colleagues [59]. The root causes of this problem are attributed to the rapid pace of urbanization, changes in land use, and industrialization, especially in countries with high populations, such as India and China. Whereas in parts of Asia and Africa, inadequately treated wastewater, effluent, and sludge used for irrigation are the main sources of plant contamination, sources of heavy metals in food plants grown in America, Europe, and Oceania arise predominantly from particulate matter (from industrial and transport sectors) and agricultural practices. Sludge from distilleries and the chemical, electroplating, textile, and leather industries is often found to contain significantly high concentrations of heavy metals, such as Cr, Cu, Fe, Mn, Ni and Pb. Gold mining is a leading source of heavy metal contamination (especially Cd, Hg, Pb) in soil and food crops. Plants used in Asian herbal medicines acquire metal contaminants (e.g., As, Cd, Cr, Cu, Fe, and Pb) when grown near smelting or other industrial areas [60,61,62].

Certain plant species can immobilize metals in soil and groundwater through absorption and accumulation by roots, adsorption onto roots, or precipitation within the root zone, a process known as phytostabilization. Other species absorb and hyperaccumulate metals/metalloid contaminants through a phytoextraction process. Several plants, such as *Brassica* spp., can accumulate lead in concentrations >50 mg/g dw. The roots of *B. juncea* (Oriental Mustard) concentrated mercury 100-270X (dw) above initial solution concentrations, but only 0.7–2% was translocated to the shoots [63]. Numerous metals have been measured in plants used for cereals, fruits, and vegetables; for example, samples of rice (*Oryza sativa*) contained chromium (15–465 mg/kg), manganese (61–356 mg/kg), and lead (16–16,500 mg/kg), and high levels of arsenic have been found in rice and lettuce (*Lactuca* *sativa*) [59].

## 6. Heavy Metals in Plants with Potential Neurotoxicity

### 6.1. Grasspea

**Figure 2:**
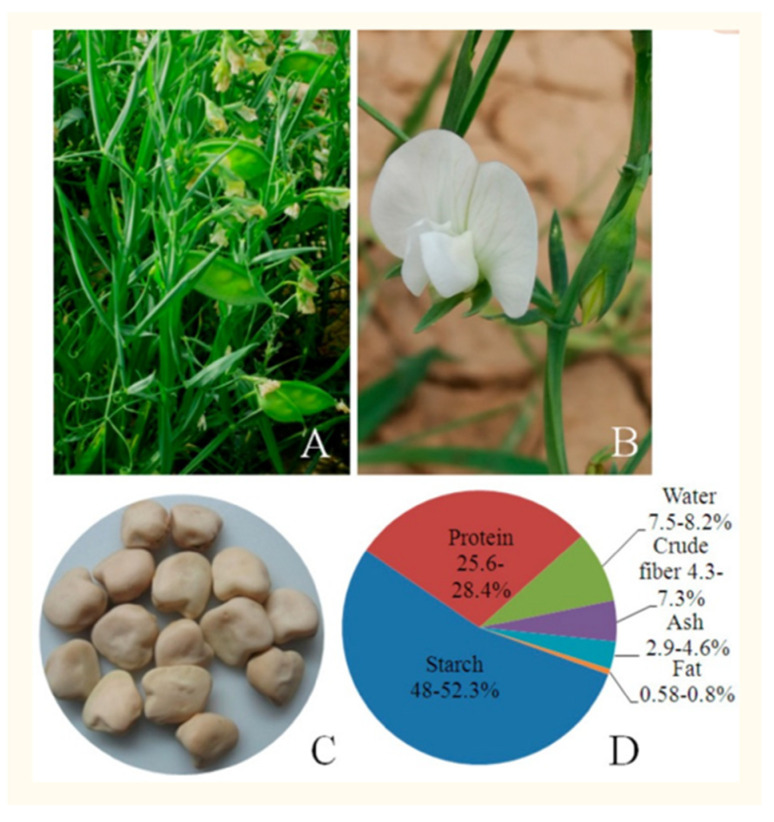
*Lathyrus sativus* (grasspea), an annual legume cultivated in arid and semiarid areas, has attractive flowers (**A**,**B**) and yields nutritious seed (**C**). The seeds are a rich source of protein and starch (**D**). Reproduced with permission from Xu et al. [68], doi:10.3390/ijms18030526, accessed on 23 February 2021.

The legume *Lathyrus sativus* (grasspea) (Figure 2), prolonged heavy consumption of which triggers the irreversible central motorsystem disease lathyrism [64,65], is a strong metal accumulator of lead and cadmium in all parts of the plants [66,67,68]. Root tissues of lead-exposed grasspea showed a six-fold, two-fold, and three-and-a-half-fold reduction in calcium, zinc, and copper contents, respectively, which indicated the plant tolerates a deficiency in essential nutrients while storing large amounts of lead [69]. The uptake of cadmium and copper in grasspea shoots was exponential over the range of concentrations tested [70]. The distribution of lead in *L. sativus* has a selective character: leaves > roots > stems > seeds. While lead is also found in the seed, soil pollution with heavy metals does not affect seed quality, such that the high nutrient (K, O, Cu, Fe, Mn, Zn) and protein content (23.18–29.54%) is preserved [71]. Grasspea can also bioaccumulate arsenic in roots > shoots [72]. These properties are important because grasspea is considered an ideal crop for resource-poor farmers, is widely eaten on the Indian subcontinent, and serves as both a regular and famine food in the northern Ethiopian highlands [73,74]. 

**Figure 3:**
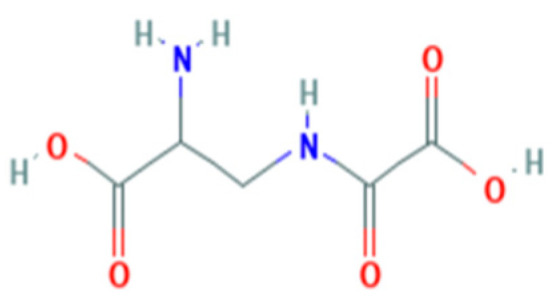
Structure of β-*N*-oxalylamino-L-alanine. Source: PubChem, National Institutes of Health National Library of Medicine https://pubchem.ncbi.nlm.nih.gov/, accessed on 14 March 2021.

The neurotoxic property of grasspea is attributable to its content of the excitotoxic nonprotein amino acid β-*N*-oxalylamino-L-alanine (L-BOAA, *syn*. dencichine) (Figure 3), also known as β-oxalyldiaminopropionic acid (β-ODAP) and, perhaps, also to its low content of methionine and cysteine [68]. L-BOAA/β-ODAP is found in all parts of the plant, with concentrations of 0.5–2.5% in traditional varieties of grasspea [75]. Zinc deficiency and oversupply of iron to the roots of *L. sativus* induce increases in the L-BOAA/β-ODAP content in ripe seed [76]. The biosynthesis of L-BOAA/β-ODAP and its genetic and environmental regulation are under intensive study because grasspea is tolerant of drought and waterlogging, flourishes without inputs, and the seed has significant protein content, such that grasspea varieties with low-L-BOAA/β-ODAP content may potentially serve as a safe and valuable foodstuff [68,77,78]. However, environmental factors such as drought, zinc deficiency, iron oversupply, and the presence of heavy metals in the soil reportedly can considerably increase the level of L-BOAA/ß-ODAP in the seed of grasspea [79].

The addition of arbuscular mycorrhizal fungi (AMF) to grasspea seedlings promoted plant growth of grasspea under sulfate stress [80]. AMF are associated with most terrestrial plants: they penetrate the roots and form symbiotic relationships that protect the plant from diverse stressors, including resistance to temperature extremes, drought, waterlogging, salinity, and heavy metals [81]. *L. sativus* is highly tolerant of such environmental stressors, which suggests AMF are normally associated with the plant and thus able to provide some protection against heavy metals. The subject is of importance to the health of people with dietary reliance on grasspea in countries such as Bangladesh, Ethiopia, India, and Nepal [77].

### 6.2. Cassava

**Figure 4:**
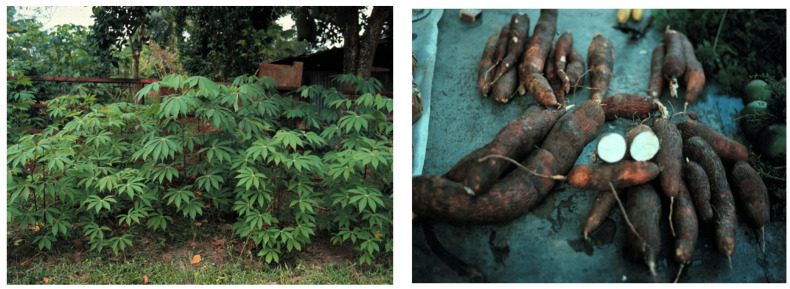
*Manihot esculenta* Crantz. leaves (**left**) and tubers (**right**).

The tubers and leaves of *Manihot esculenta* (cassava) provide a source of food for huge numbers of people in Asia, Africa, and South America (Figure 4). For example, cassava tubers supply ~70% of the daily calorie input for >50 million people in Nigeria, the world’s largest cassava producer (49 million metric tons/year). Fresh cassava root contains >30% carbohydrate, >2% protein, 0.1% fat, and >75% moisture content [82,83]. However, *M. esculenta* also synthesizes cyanogenic glucosides (linamarin and its methylated relative lotaustralin; 97:3), the concentration of which must be substantially reduced before the tuber is used for human consumption. The method of processing cassava roots, the duration of tuber storage, and the type of meal preparation determine the amount of consumer exposure to cassava cyanogens and heavy metals [84]. Failure to remove the cyanogenic glucosides and their products before human ingestion can precipitate acute cyanide (HCN) poisoning or, with continued daily consumption, the subacute onset of an irreversible motorsystem disease (cassavism), which is known as *konzo* in the Democratic Republic of Congo and *mantakassa* in Mozambique [85,86]. Whether the daily consumption of cassava-derived *gari* in Nigeria is also responsible for endemic peripheral neuropathy with optic atrophy and sensorineural deafness has been questioned.

Acute toxicity from cassava processing and/or ingestion results from cyanide ion (CN^–^) binding to iron and copper sites in cytochrome *c* oxidase, which is required for electron transport and energy generation. Cassavism is associated with elevated levels of urinary thiocyanate (SCN^–^) produced in the consumer by the addition of endogenous methionine/cystine sulfur to cyanide by thiosulfate sulfurtransferase (rhodanese) [87], the enzyme activity of which is inhibited by certain metal ions (Au, Ni, Pt, and Zn) [88]. Laboratory animals given a sulfur-free diet also eliminate CN as cyanate (OCN^–^) [89], prolonged exposure to which (in the form of NaOCN) can precipitate peripheral neuropathy [90]. All three anions (CN^–^, OCN^–^, and SCN^–^) form metal complexes, at least in the chemistry laboratory [91,92,93]. Cyanide also forms stable complexes with cobalt, gold, iron, and mercury, a property used commercially to leach the metal from gold-bearing ore [94]. Linamarin may be involved in the uptake of gold and mercury into cassava roots [95].

Several studies have shown heavy metals in cassava and the processed flour derived from its root. Cassava in communities in Nigeria’s River State contained metal elements in both the leaves (Fe > Cu > Cr > Ni > Pb > Cd) and tubers (Fe > Cu > Cr > Ni > Pb > Cd) [96]. Metal concentrations in the tubers (Cd, Ni, and Pb) and leaves (Cd, Cr, and Pb) of cassava plants grown on soils around a lead sulfide (galena) mining area in Nigeria’s Ebonyi State exceeded WHO standards [97]. Exceedances for Al, As, Ca, Cr, Cu, Fe, K, Mn, Pb, and Zn were found in cassava tubers growing around two cement factories in Ogun State, Nigeria [98]. Cassava grown on soil contaminated with crude oil in the Niger Delta Region contained heavy metals (Fe > Zn > Ni > Pb > Cd > Cr) in their leaves and tubers [99].

Automobile emissions added to the metal content of soil samples and cassava plants collected from farmlands along a major expressway in Nigeria’s Delta State [100]. Mean levels (mg/kg) of heavy metals (Fe> Zn> Ni> Cr > Pb) in soil samples were 142.93 ± 42.16 for Fe, 59.34 ± 25.21 for Zn, 24.98 ± 15.57 for Ni, 14.27 ± 5.39 for Cr, and 13.63 ± 5.41 for Pb. Corresponding mean metal concentrations (mg/kg) in cassava leaves and tubers were, respectively, 21.70 ± 3.45 and 9.62 ± 3.53 for Fe, 4.15 ± 1.01 and 1.15 ± 0.44 for Zn, 5.12 ± 2.75 and 0.37 ± 0.63 for Cr, and for leaves only, 3.46 ± 1.58 for Pb. Plant concentration factor values corresponded to Cr > Pb > Fe > Ni. Cassava harvested from farmlands along highways in Owerri, Nigeria, contained leaf concentrations of Zn > Cu >Pb> Ni > Cd [101].

Metal contamination also occurs from the common practice of sun-drying cassava flour by the roadside [102]. Significantly elevated concentrations of Pb > Fe > Cu > Cr > Mn > Zn > Ni > Cd > Co were found in cassava flour processed by roadside drying along a Nigerian highway [103]. Samples of cassava flour purchased from the capital city of Nigeria’s Osun State contained lead concentrations (up to 0.34 mg/kg) that far exceeded the Nigerian Industrial Standard Permissible Level (0.1 mg/kg). However, flour levels (mg/kg) of CN (0.03–0.09), cadmium (0.01–0.0), copper (0.35–0.62), iron (0.1–0.6), nickel (0.20–0.49), selenium (3.46–5.43), and zinc (0.182–0.3) were within permissible limits [104].

While several studies have revealed the potential for heavy metal contamination of cassava roots and leaves used for food in sub-Saharan Africa, whether and which metals can influence the content, speciation, or neurotoxic potential of the plant’s cyanogens and their metabolites are unknown. Metal neurotoxicity has not been reported or looked for in those who use cassava as a food staple.

## 7. Key Conclusions

Botanicals used for food, nutritional supplements, and medicinal purposes can bioaccumulate metals/metalloids with neurotoxic potential in concentrations that, in some circumstances, exceed permissible limits for human consumption. While certain heavy metals/metalloids, such as arsenic, lead, manganese, and mercury [105,106,107,108], have established human neurotoxic potential, especially during brain development, their effects from ingestion of contaminated botanicals have rarely if ever resulted in diagnosed neurological illness. Associations have been reported between heavy metals and certain neurodegenerative diseases, including amyotrophic lateral sclerosis [109,110], Parkinson’s disease [111,112], and Alzheimer’s disease [113,114], but evidence of cause–effect relationships is generally lacking. Chemical principles in some mushrooms (hydrazone/hydrazines) and plants (dencichine, cyanogens) have established human neurotoxicity [115,116], their concentration varying in response to the uptake of metal elements from soil and water. Such botanical species normally pose serious health hazards to humans because of their potential for acute, chronic, and possibly long-latency neurological disease, the risk for such effects varying with the amount and duration of consumption. Witness the extraordinarily high prevalence (5.5% in 2019) of spastic paraparesis (lathyrism) in northeast Ethiopia, with somewhat higher rates (6.2%) among those who use traditional iron-containing clay pots to cook grasspea [117]. The metallic content of botanicals used for food, thus has the potential for direct and indirect adverse effects on the human nervous system. On the other hand, the ability of certain botanicals to bioaccumulate certain metals/metalloids is exploited to protect human health by reducing soil and water pollution via phytoremediation and mycoremediation [118,119].

## Data Availability

All data were drawn from published works as cited.
